# ExNOTic: Should We Be Keeping Exotic Pets?

**DOI:** 10.3390/ani7060047

**Published:** 2017-06-19

**Authors:** Rachel A. Grant, V. Tamara Montrose, Alison P. Wills

**Affiliations:** 1 Animal Behavior and Welfare Research Group, Department of Animal and Agriculture, University Centre, Hartpury, Gloucestershire GL19 3BE, UK; Tamara.Montrose@Hartpury.ac.uk; 2 Animal Health Research Group, Department of Animal and Agriculture, University Centre, Hartpury, Gloucestershire GL19 3BE, UK; Alison.Wills@Hartpury.ac.uk

**Keywords:** exotic pets, parrots, degus, guinea pigs, rabbits, rodents, amphibians, reptiles, welfare

## Abstract

There has been a recent trend towards keeping non-traditional companion animals, also known as exotic pets. These pets include parrots, reptiles, amphibians and rabbits, as well as small species of rodent such as degus and guinea pigs. Many of these exotic pet species are not domesticated, and often have special requirements in captivity, which many owners do not have the facilities or knowledge to provide. Keeping animals in settings to which they are poorly adapted is a threat to their welfare. Additionally, owner satisfaction with the animal may be poor due to a misalignment of expectations, which further impacts on welfare, as it may lead to repeated rehoming or neglect. We investigate a range of commonly kept exotic species in terms of their suitability as companion animals from the point of view of animal welfare and owner satisfaction, and make recommendations on the suitability of various species as pets.

## 1. Introduction

A pet can be defined as an animal kept for companionship or pleasure. There has been a trend in recent years towards keeping pets other than traditional domesticated species such as dogs and cats [1]. Dogs have been associated with humans for thousands of years, and through artificial selection have become well adapted to life as a human companion or worker [2]. Cats are commensal and retain more natural behavior, but again, there has been a long and mutually beneficial relationship with human beings [3]. Dogs and cats are not generally confined to small enclosures, information about their care and welfare is plentiful, and there are numerous veterinary practices that have the expertise and facilities to treat them [4]. In the past two decades, non-domesticated species of pets such as reptiles, exotic mammals (e.g., degus), amphibians and exotic birds (usually parrot species) have become popular as pets [5]. These pets are not always easy to care for as they may retain more wild behavior than, for example, dogs and cats, which are adapted to live with humans. While dogs and cats do exhibit behavioral problems and are not always treated in ways conducive to optimum welfare, they are not fundamentally unsuitable as pets, and large amounts of information is available on their proper care. In contrast, many exotic pets have specialized requirements in captivity that are beyond the scope of many pet keepers to provide [6,7,8,9]. Even some pets that have traditionally been seen as good children’s pets, such as rabbits and small rodents, may actually provide poor owner satisfaction in the long term due to innate behaviors that may be misaligned with owner expectations [7]. The welfare of these exotic pets is often at risk through a combination of factors, including a lack of accurate information available on their care, incorrect husbandry, and the unrealistic expectations of owners. This is often compounded by a lack of specialist veterinary care [4] and a lower propensity for owners to avail themselves of such care [10]. In this commentary, we review the suitability of a range of exotic species, from the point of view of animal welfare and owner satisfaction, and make recommendations on which taxa can make suitable companion animals.

## 2. Parrots and Cockatoos

Parrot species (Aves: psittaciformes) differ in their suitability as pets. Large parrots (e.g., *Amazona* spp.), macaws and cockatoos are highly intelligent, have a long lifespan and often exhibit neurosis-like personality traits and a predisposition to stereotypy and abnormal behavior indicative of poor welfare states [8,9,11,12,13,14]. This means that it would be difficult to provide for their needs in captivity, and as companion animals they are likely to suffer reduced welfare to some extent [8,9,14]. African Grey parrots (*Psittacus erithacus*) have been the subject of several studies on intelligence, cognition and referential communication, [15,16,17,18] and providing for their advanced cognitive and social needs can only be achieved by the most dedicated of pet keepers. Amazon parrots (*Amazona* spp.) have also been shown to suffer poor welfare when caged without appropriate enrichment [19,20]. Unsurprisingly, the aforementioned large parrot species are also the most prone to stereotypic feather mutilation, an indication of psychological distress and poor welfare [8,9,21,22]. The larger parrots and cockatoos also have a long lifespan (up to 80 years), meaning they may need to be rehomed several times during their lives [9].

Pet parrots vary in the extent of their domestication, with most being either wild caught or first or second generation [9,23]. There is an illegal trade in wild parrots that continues to cause significant welfare issues during capture, transport and at the eventual destination [24,25]; therefore, keeping wild-caught parrots is unethical and is not recommended for any reason. First or second generation captive bred parrots cannot be considered domesticated and are genetically identical to wild parrots; as such, their ethological needs coincide with those of wild birds [9]. Wild parrots spend most of their time flying, foraging and interacting with conspecifics [26], and although there are species-specific differences in behavior, their needs in captivity are broadly similar. In the authors’ opinion (based on many years of keeping parrots and research into their behavior), the major welfare concerns in pet parrots are social isolation, flight restriction, poor diet (including lack of foraging enrichment) and hand rearing (which is effectively social, parental and filial deprivation).

Most wild parrots are highly social and these prey species are protected from predation by flocking (through predator dilution and vigilance) [9,26]; therefore social isolation is likely to cause severe psychological distress. The flock is important not just for protection from predation but also for mate choice, communal foraging, allogrooming, and offspring socialization. Several studies have found solo housing to be linked to stereotypic behavior and poor welfare, and there is evidence that parrots suffer less when kept in pairs or groups [27,28].

Flight restriction occurs when birds are confined to a cage and/or when wings are clipped. Over-caged birds (i.e., those kept for 10 or more hours per day in the cage) are likely to suffer and exhibit abnormal behavior such as repetitive locomotion and bar biting, which has been directly linked to barren cage environments [20]. It has been estimated that 50% of pet parrots are kept in enclosures that are too small to promote adequate welfare [9]. Based on their natural history, parrots have ethological requirements for space to fly and social interaction, and the authors recommend that psittacines need a minimum of 4–6 hours of daily flight time out of the cage, preferably socializing with other parrots. Owners may want to consider housing psittacines in indoor or outdoor aviary-style accommodation instead of a cage. Wing clipping, as well as being a threat to welfare, is also unnecessary as birds can easily be trained to obey most requests from their owners [29,30]. Although safety is cited as the main justification for wing clipping [31], wing-clipped birds may be less safe, as they are unable to escape from danger. Wing clipping also deprives parrots of a source of exercise and the ability to carry out natural and highly motivated behavior [30]. Removing an animal’s main method of escape from danger could cause them to suffer negative mental states, such as fear [30]. Expression of normal behavior (such as flight) is a criterion for adequate welfare; therefore, we believe that wing clipping is generally undesirable, but must be considered on a case-by-case basis.

Parrots also have specialized dietary needs, which many owners are not aware of. Captive diets consisting of all-seed traditional parrot mix are nutritionally inadequate [32]. Furthermore, appropriate levels of enrichment, in particular foraging enrichment, are not always provided for pet parrots, which can cause abnormal behavior and impaired welfare [9,20].

Hand rearing is the practice of deliberately raising and feeding the parrot chick away from its parents and other conspecifics and is mainly done to increase the tameness of the parrot, and make it imprinted onto humans and more dependent on human companionship [33]. Parrots are flock animals that learn social skills through extensive interaction with conspecifics and these “cuddly tame” hand-raised parrots that have not had this early experience are in demand by pet owners. However, on reaching sexual maturity they do not behave normally [34], and may be more interested in human companionship than that of other psittacines [35]. Williams et al. [28] found that hand-reared parrots in a zoo setting showed more stereotypy and less interaction with enrichment than parent-reared birds, and hand rearing is also reported to cause abnormal fear and phobic reactions to develop [35]. Indeed, although hand-reared birds are preferred by owners initially, later there can be reduced owner satisfaction due to behavioral problems such as aggression, fear and unwanted sexual behavior directed towards owners [36]. The purchase of hand-reared “cuddly tame” parrots from breeders only perpetuates the welfare problems that these human-imprinted birds face in captivity. The best parrot pets, both from the point of view of the birds’ welfare and the long-term satisfaction of owners, are in the authors’ opinion likely to be parent-reared birds that have been socialized to humans through careful handling (pers obs). Warwick et al. [4] have developed the EMODE system, which scores the level of difficulty of meeting the biological needs of pets. Birds generally are scored as moderately to extremely difficult to keep, and parrot-type birds, especially those with a long life span and that have been imprinted onto humans have a high score using this method, meaning it would be difficult to fully meet their needs in captivity.

Having said that, keeping parrot-type birds provides many benefits, with owners’ perception being that they receive love, emotional support and companionship from their birds, as well as considering them a member of the family [37]. The needs of parrots are likely to vary by species, but few studies have investigated species-specific personality and behavior differences in psittacines. It is clear that the larger parrot species are fundamentally unsuitable as pets for reasons already outlined [8]. As long as the ethological needs for social interaction, space, enrichment, flight and diet are provided for, some of the smaller parrot species such as lories, lorikeets, caiques, *Pionus* and *Poicephalus* species, cockatiels, conures and budgerigars may make suitable pets [8,22].

As the smaller species are also more economical to buy and feed, it becomes easier for owners to address their social and spatial needs [38]. Having said that, the individual species must be researched fully, since for example, some conure species are extremely vocal, and lorikeets require a specialist nectar diet. Also, smaller birds may be seen as disposable because of their lower cost, and therefore the threats to their welfare may be different, yet as acute as the larger parrots [9]. Budgies, for example, being a popular pet, may be kept by owners who are not informed of the bird’s needs, so smaller parrots may suffer more from ignorance of their captive needs [14]. It is concerning that a survey by the American Veterinary Medical Association (AVMA) showed that in 2001, 11.7% of bird owners in the USA reported at least one veterinary visit, compared to 83.6% of dog owners and 65.3% of cat owners [10].

The Netherlands has banned hand-rearing psittacine birds [39], but other European countries have not yet followed suit. The UK put in place the more general Animal Welfare Act in 2006, which means that owners have a “duty of care” to allow the expression of normal behavior [40]. Wing-clipping, over-use of a cage and social isolation are clearly contrary to this, but the law is highly unenforceable, with many parrots being kept this way (pers obs) and few prosecutions occurring to date. United States legislation is inadequate at both state and federal levels [9]. Clearer, species-specific legal guidelines are required for parrots.

## 3. Reptiles and Amphibians

It is difficult to quantify the extent of herpetological pet keeping, but it is thought to be extensive and involve at the very least tens of millions of animals [6]. For example, the USA alone is thought to import two million reptiles annually, and also exports 2–4 million baby “pet” turtles, with an estimated 12 million reptiles being kept in private homes [6]. The European Union is also a large market for the reptile trade, with estimated imports of 6.7 million live animals of various species between 2005 and 2007 [6]. Many authors have expressed concern in terms of ethics and welfare about the growing trend for keeping these animals [6,41,42], which has created demand for their removal from the wild and is responsible for huge mortality and morbidity [6,41].

As well as the ethical concerns surrounding the trade of these animals, reptiles and amphibians require specialized care and do not make suitable pets. Reptiles and amphibians have species-specific thermal, hydrological, dietary and behavioral requirements, and most owners lack a basic understanding of their needs in captivity. Whilst there are a number of exceptional hobbyists who are knowledgeable and scrupulous about providing for the needs of their animals, the vast majority of pet reptiles are kept in inadequate enclosures with poor husbandry and a lack of understanding of their biological needs [43]. Toland et al. [44] estimate that 75% of reptile pets die within a year of acquisition, and although other sources report a much lower figure [42], the welfare issues in capture, transport and husbandry are still significant. The issues are multiple, but include calcium deficiency (and associated metabolic bone disease), incorrect humidity levels, trauma due to escape attempts, thermal stress, inappropriate handling, and poor diet. Unlike dogs and cats, reptiles and amphibians are usually restricted in their movements in inadequately sized enclosures [6,43]. Social isolation, however, is less of a problem with amphibians and reptiles than with other, more social taxa. In addition to the welfare threats to the animals, reptiles and amphibians often carry zoonotic diseases, primarily salmonellosis, which is a particular concern if there are children or pregnant women in the household [45,46,47]. The Internet contains numerous care sheets and other information on keeping reptiles and amphibians in captivity, but misinformation and erroneous statements abound, particularly relating to the ease and suitability of keeping the animals [6,43]. There is the perception that certain species are easy to keep, and that they may be less demanding than larger pets and require less space, none of which are supported by the available evidence [6,43]. Owners are not generally knowledgeable enough to recognize the signs of stress and poor welfare in reptiles and amphibians, and many veterinarians do not have the specialized knowledge required to treat these species [4]. There are no reptiles or amphibians that are “easy to keep” [4,6,43], and for these reasons we do not recommend these animals as suitable pets.

## 4. Rabbits and Rodents 

### 4.1. Rabbits

Rabbits are a popular pet in the UK [48] and the USA [49], with the estimated pet population ranging from 0.8 to 1.2 million rabbits in the UK alone [48,50] and three million in the USA [49]. Rabbits are also becoming popular companion animals in Mediterranean countries such as Spain, where traditionally they have been kept for meat or fur [51]. Despite their popularity, rabbits are not always kept appropriately, with owner knowledge of correct housing, preventative medicine, diet, handling and their pet’s behavioral needs frequently being lacking [50,52].

The UK’s Rabbit Welfare Association & Fund (RWAF) [52] recommends that rabbits be housed in hutches of a minimum size of 1.83 m × 0.6 m × 0.6 m (with a floor area of 1.10 m^2^) which should be attached to a secure run of at least 2.44 m × 1.83 m (4.5 m^2^) [52]. These dimensions are proposed to allow rabbits to move, stand up and separate feeding, resting and excretion areas [52]. It has been reported that smaller enclosures (0.6 m × 1.47 m/0.88 m^2^), equivalent to a standard rabbit hutch size, have been found to inhibit rabbits’ behavioral repertoire, with greater inactivity and less interaction with environmental objects shown compared to those housed in larger enclosures (2.28 m × 1.47 m/3.35 m^2^) [53], which supports the RWAF’s recommendations. A survey of the English population by Rooney et al. [54] recently found that 27.5% of rabbits were kept in enclosures smaller than 0.88 m^2^, as well as only 23.5% of rabbits having continual access to a run. These are clear areas of concern considering the RWAF recommendations [52] and the detrimental welfare effects of restricted enclosures [53].

Rabbits commonly contract a range of diseases including dental disease, gastrointestinal diseases, skin conditions and myiasis (fly strike) [55,56,57]. Many of these issues can be addressed if they are detected early on via health checking by owners. The UK Royal Society for the Prevention of Cruelty to Animals (RSPCA) [58] advises daily general health checks and more thorough weekly checks, but currently the prevalence and frequency of rabbit health checking by owners is unknown. Myxomatosis and Rabbit Haemorrhagic Disease are usually fatal to rabbits, and also result in pain and suffering prior to death [59]. Yearly vaccination is advised [59,60]; however, 30–52% of owners have not vaccinated their rabbits against these diseases [50,54,60].

Rabbits should have a diet predominantly consisting of hay [61,62]. Rabbit muesli should be avoided due to concerns regarding selective feeding, obesity and dental disease [63,64,65]. Within the UK, whilst the majority of owners feed their rabbits hay, fresh greens or pellets, 32.5% still feed rabbit muesli (all of which is not eaten in 52% of cases) and 10% do not feed hay on a daily basis [54]. This is a clear concern, as the diet that the rabbit receives can influence the development of dental disease and obesity, as well as diseases such as myiasis [64,65,66].

In the wild, rabbits are prey to many other animals, and this can be an important consideration when handling pet rabbits. Rabbits should be approached and picked up in a non-threatening manner, ideally not from above in order to avoid inducing fear [67]. Full support of rabbits when handling is necessary to help avoid stress in the rabbit and prevent falling [68]. Correct handling and restraint is also important to avoid back injuries in rabbits [69]. Unfortunately owners may use inappropriate handling techniques, which induce stress or provide inadequate support. Rooney et al. [54] report that the majority of rabbits (61%) do not respond calmly when handled by their owners, suggestive that this handling may be causing stress to the animals.

Wild rabbits live in large social groups, are very active [61], dig extensive warrens [7] and have relatively large home ranges [70,71]. Domestic rabbits display similar behaviors to wild rabbits [72,73] and are likely to have similar behavioral needs. Despite recommendations that rabbits should be socially housed [68,74,75], 57–58% of rabbits are kept alone in the UK [50,54]. Issues can arise from solitary housing, such as abnormal behaviors [76] and a reduced lifespan [77]. In addition, whilst there are a number of studies documenting the welfare benefits of providing rabbits with environmental enrichment such as gnawing sticks and boxes [78,79], and clear husbandry advice regarding this provision [80,81], many rabbits’ behavioral needs are not met. The People’s Dispensary for Sick Animals [50] reports that only 49% of rabbits get daily play with toys, 46% get play in the run, 40% get play in the garden, and 24% get opportunities for daily digging. Meeting the behavioral needs of rabbits is crucial to avoid abnormal behaviors and behavioral problems, and enhance their welfare [76,79,81].

Whilst there are a number of concerns associated with keeping domestic rabbits, they are not fundamentally unsuitable as pets as long as potential owners research rabbits and appropriately consider their health and husbandry needs.

### 4.2. Degus

Degus (*Octodon degus*) are social, long-lived, diurnal rodents native to Chile [82], although most pet animals are captive bred. As with all species, requirements in captivity reflect wild behavioral ecology. The RSPCA considers degus vulnerable in captivity because of their specialist needs [83]. In particular, they are susceptible to heat stroke. In the wild they live in the Andes, sometimes at very high altitude, so their enclosure needs to be maintained below 20 °C. They also need to be kept away from draughts, as they are susceptible to respiratory disease. As a prey species, degus may suffer fear of being swooped on from above, so a solid cage top is recommended. For the same reason, degus may not rate highly on owner satisfaction, particularly as a children’s pet, as they do not like to be handled and doing so will cause them stress. Degus are highly social [84], and, like parrots, rely on vigilance and the collective detection of predators [85], so should not be kept singly. Degus also require a specialized diet low in sugar to prevent diabetes. A study of 300 degus presented to a veterinary clinic found that most disease in the species was caused by poor husbandry and handling, including acquired dental disease, alopecia caused by fur chewing (self-mutilation), cataracts, trauma, diabetes mellitus, and hyperthermia [86]. It was concluded that owners’ knowledge levels were not sufficient to properly care for their animals in most cases. Degus may be suitable companion animals, but only for someone willing and able to devote significant time and resources to learn about and cater for their complex requirements. We particularly do not recommend degus as family pets; other rodent species may be more suitable as pets for families with children.

### 4.3. Guinea Pigs 

Guinea pigs are popular pets, with 0.7 million kept in the UK and 1.36 million in the USA, making them the second most common small mammal after rabbits [87,88]. Guinea pigs are also often selected as pets for children due to their placid, docile temperament and ease of handling. As guineas pigs are socially tolerant animals [89], most reputable pet stores will only sell guinea pigs in pairs to avoid welfare concerns associated with social isolation.

Although not strictly classed as exotic pets, guinea pigs have specific husbandry requirements that differ from those of other rodent species and rabbits. When their physiological and behavioral needs are adequately met, guinea pigs can make rewarding pets that are neither expensive nor difficult to keep. Guinea pigs require a dietary source of Vitamin C; however, a number of recent studies have reported that owners are aware of this and many supplement their animals in addition to providing dietary materials high in Vitamin C [90,91]. Similarly to rabbits, guinea pigs require a high fiber diet in order to maintain good gastrointestinal health and avoid gastrointestinal stasis [92]. Owners need to be aware that guinea pigs require constant access to high quality hay in order to prevent the development of disease. Other common issues that affect guinea pigs include dental disease, ocular disorders and ectoparasitic infections [91].

Whilst some of the common disorders affecting guinea pigs are relatively simple and cost effective to treat (e.g., parasites), others such as dental disease require ongoing treatment and management [93] that owners may be unwilling to financially invest in. This is particularly the case when affected animals have a guarded prognosis, which may lead owners to believe that euthanasia is the best option. Problems such as dental disease are most successfully treated when diagnosed early, but when guinea pigs live in an outdoor hutch with conspecifics, it may be difficult for owners to identify subtle signs, such as a decrease in food consumption [94]. Further issues include the lack of confidence of some veterinarians in diagnosing and treating dental disease in guinea pigs [91,95]. It has been reported that a lack of dietary fiber is the primary cause of dental disease in rodents [94]; however, the only experimental study to investigate this in guinea pigs failed to relate tooth wear to dietary abrasiveness [96]. It is recommended that guinea pig owners consult a veterinarian experienced in treating exotic pets if their animals show signs of ill health.

Many of the health problems that affect guinea pigs occur in older animals [97]. By this point children may have lost interest in their pets, and if animals are not routinely handled, signs of ill health may not be observed. The incidence of oral cavity disease is higher in older animals [98] and geriatric males often suffer impaction, which may require daily owner intervention to manage.

Guinea pigs are also susceptible to respiratory tract infections, but the reported prevalence varies quite considerably. It has been identified that the development of pneumonia is linked to housing animals in damp or dusty conditions [99]. Pathogenic causes of respiratory disease include viruses and bacteria, but mortality rates are high regardless of the etiological agent [100]. The RSPCA recommend that in temperatures below 15 °C, guinea pigs are moved indoors unless their outdoor accommodation is suitably insulated. There is limited research on whether owners prefer to house their guinea pigs indoors or outdoors, but it could be hypothesized that an indoor environment protects against the fluctuations in temperature that can lead to the development of disease. It would seem likely that most outdoor-housed guinea pigs are not routinely brought indoors when the weather is colder. Unlike rabbits, guinea pigs are not readily litter trained, which means that they cannot be kept as house animals. However, guinea pigs still require space to exercise and their welfare may be compromised if they are predominantly left in their hutch or enclosure. The floor of guinea pig cages should be smooth and solid as housing guinea pigs on wire mesh can cause injury to the feet and subsequent pododermatitis [101].

Behavioral problems are uncommon in guinea pigs [89]; therefore, these seem an unlikely cause of owner dissatisfaction. Bonding mature males can be challenging, but this can be easily overcome by keeping neutered males with females or by introducing same sex pairs when young.

We recommend that guinea pigs can make good pets if they are kept by interested adults who are aware of their potential lifespan and husbandry requirements. Whilst the temperament of guinea pigs makes them a good pet for children, it is imperative that an adult takes responsibility for their welfare and appreciates the potential costs associated with health problems that they may experience in later life.

## 5. Conclusions

In conclusion, whilst there are increasing numbers of exotics being kept as pets, this unfortunately does not reflect increased public understanding of their needs. The difficulties of keeping these animals are also often underestimated by owners. Whilst some exotic animal species such as budgerigars, parakeets, rabbits and guinea pigs are likely to be suitable as pets as long as owners conduct research into their lifespan, husbandry, ethological and health requirements, meeting the needs of exotic pets such as large parrots, reptiles and amphibians is likely to be challenging in captivity. In particular, we feel that the lack of accurate and comprehensive information on keeping these pets and the difficulty in finding specialist veterinary care for exotics puts them at risk of both behavioral and physical problems. Whilst exotics are not the only pets that can suffer welfare problems when not properly cared for, there is much more information available on the needs and proper care of cats and dogs. This stands in stark contrast to the situation for many exotic pets, and we believe that not only careful research and planning, but also much greater consideration of whether an exotic animal should be kept as a pet at all, is needed before owners acquire them.
